# The analysis of the SIRS alcoholism models with relapse on weighted networks

**DOI:** 10.1186/s40064-016-2308-0

**Published:** 2016-06-14

**Authors:** Hai-Feng Huo, Ying-Ping Liu

**Affiliations:** Department of Applied Mathematics, Lanzhou University of Technology, Lanzhou, 730050 Gansu People’s Republic of China

**Keywords:** Alcoholism, Threshold, Fixed weight, Adaptive weight, Optimal control, Stable

## Abstract

Two SIRS alcoholism models with relapse on networks with fixed and adaptive weight are introduced. The spread of alcoholism threshold ${R_0}$ is calculated by the next generation matrix method. For the model with fixed weight, we prove that when ${R_0} < 1,$ the alcohol free equilibrium is globally asymptotically stable, then the drinking crowd gradually disappear. When ${R_0} > 1$, the alcoholism equilibrium is global attractivity, then the density of alcoholics will remain in a stable value. For the model with adaptive weight, we only make some numerical simulations. We also give two effective strategies. Our results show that the treatment of recuperator for stopping relapsing and preventing the susceptible people to drink are two effective measures to eliminate alcoholism problem, and preventing the susceptible people to drink is more effective when the proportion of recuperator to accept treatment is equal to the proportion of susceptible people to refuse drinking alcohol.

## Background

Alcohol use and abuse have been part of human society for centuries. The College Alcohol Study defines alcoholism as male students who had five or more and female students who had four or more drinks in a row at least once in a 2-week period (Wechsler 2000). Similar to other drug addictions, alcoholism can cause to a series of serious consequences. The World Health Organization estimates that about 140 million people around the world under the influence of alcohol-related problems, such as being sick, losing a job and so on (Saunders et al. 1993). What is more serious is that chronic alcohol consumption damages almost all parts of the body and contribute to a number of human diseases including but not limited to liver cirrhosis, pancreatitis, heart disease, and sexual dysfunction and eventually be deadly (Glavas and Weinberg 2005). Early recognized since the 1800s that alcoholism produced not only impairment of the senses but also higher predisposition for tuberculosis. William Osler reported in 1905 that patients who misused alcohol had higher predisposition to pneumonia (Giraldina et al. 2015). And studies over the last 30 years have also demonstrated that chronic alcohol consumption impairs the functions of both T cells and B cells (Sumana et al. 2015). Between 2006 and 2010 in the United States, alcohol abuse resulted in approximately 88,000 deaths, and the average death rate associated with alcoholism was 28.5 per 100,000 population (Gonzales et al. 2014). Importantly, the majority of alcohol-related deaths were among adults aged 20–64 years old (Giraldina et al. 2015). In view of the above situation, alcoholism has become a issue that need to be solved urgently.

Since mathematical model can mimic the process of alcoholism and provide useful methods to control the spread of drinking behavior. Several different mathematical models for alcoholism have been formulated and studied recently. Sanchez et al. (2007) presented a simple model for alcohol treatment. Their model were based on studying binge drinking in a college system and assumed the same “leaving rate”. Manthey et al. (2008) built a model to capture the dynamics of campus drinking and to study the spread of drinking on campus. Benedict (2007) proposed an SIR model and used standard contact rate between susceptible and alcoholics. Furthermore they obtained the alcoholism reproductive number and discussed the existence and stability of all the equilibria. Huo and Song (2012) introduced a two-stage model for binge drinking problem, which the youths with alcohol problems were divided into those who admit the problem and those who do not admit it. Wang et al. (2014) presented a deterministic SATQ-type mathematical model for the spread of alcoholism with two control strategies and analyzed some properties of the solutions including positivity, existence and stability. Huo and Wang (2014) developed a nonlinear mathematical model with the effect of awareness programs on the binge drinking. Their results showed that awareness programs is an effective measure in reducing alcohol problems.

Quit drinking is usually temporary. Some drinking people may relapse since contacting with alcoholics or weak self-control ability. Sharma and Samanta (2015) developed an alcohol abuse model by introducing a treatment programm in the population and considered all possible relapses. They assumed that the drinkers in treatment most commonly relapse due to contact with heavy drinkers who are not in treatment. For the other mathematical models for alcoholism or smoking, please see Wechsler and Nelson (2008), Room et al. (2005), Mushayabasa and Bhunu (2011), Huo and Zhu (2013) and references cited therein. They commonly assume that communities are homogeneous, that is, communities are made up of individuals who mix uniformly and randomly with each other in the above models. These assumptions make the analysis tractable but not realistic (Bansal et al. 2007).

In contrast to classical compartment models, a lot of studies on complex networks have been investigated during the past years. Liu et al. (2013) presented an SIR model with individual’s birth and death on scale-free networks and analyzed the stability of three equilibria. They also gave out two immunization schemes. But they didn’t consider recuperator’s temporarily immune, that is to say, recuperator is likely to become infected or susceptible because of the loss of immune. Zhu et al. (2012a) investigated a new epidemic SIS model with nonlinear infectivity on heterogeneous networks. The global behavior of the model is studied. Wang et al. (2012) proposed a modified SIS model with an infective vector on complex networks. They treated direct human contacts as a social network and assumed spatially homogeneous mixing between vector and human populations. Huo and Liu (2016) proposed an alcoholism model on complex heterogeneous networks and proved stability of all the equilibria.

Nodes usually represent individuals and links represent potential contacts among those individuals on the complex network (Zhu et al. 2012a). The connectivity of a node is defined as the number of the links connected to the node, represent by *k*. The degree distribution of a network is defined as the probability of a randomly chosen node to have a degree *k*, represent by *P*(*k*). Many networks (Zhu et al. 2012b; Liu and Zhang 2011; Zhang and Jin 2011; Liu et al. 2004) have been found to be scale-free networks, that is to say the degree distribution follows a power law distribution $P(k)= c{k^{ - \gamma }}, (2 < \gamma \le 3)$, where *c* is any constant satisfy the equation $\sum \nolimits _{k = 1}^n {P(k)} = 1$.

Notice that in many real contact networks, there are groups of nodes with a high density of edges within them and a lower density of links between groups. The differences between links within a contact network can be described by link weights, which can represent the amount of time two individuals interact or the intimacy between individuals. The larger the weight is, the more the two nodes communicate, while, the more possible a susceptible individual will be infected through the edge (Chu et al. 2009). The usual assumption is that weights are constant and driven by the network connectivity, which is fixed as time goes on. For example, the weight between two nodes with degrees *i* and *j* are represented by a function of their degrees (Barrat et al. 2004a, b, c). However, as the disease becomes severe, individuals tend to be more cautious in social contacts and make some reflection such as decreasing the out going visits, cutting down the meeting time and reducing the intimacy. Such behaviors will change the strengths of nodes and the weights of links, which can be seen as an adaptive weight network. Further, it was found that the infectivity exponent has a stronger effect on the epidemic threshold and the epidemic prevalence than the weight exponent (Chu et al. 2011). For the other mathematical models on network with weights, please see Macdonald et al. (2005), Zhu et al. (2013) and references cited therein.

Motivated by the Liu et al. (2013), Zhu et al. (2012b, 2013), we introduce the individuals’ birth and death in our model, and set up an SIRS alcoholism model in complex network. Furthermore, we study the impact of the fixed weight and adaptive weight on the spread of alcoholism. We not only introduce general forms of the weight function to account for different cases of transmission but also to analyze the influence of weights on alcoholism spreading. In addition, we add the group of recuperator and study the relapse of the recuperator. We also give some control strategies against drinking, our results show that the treatment of recuperator for stopping relapsing and preventing the susceptible people to drink are two effective control strategy, and the latter has more effective than the former when the proportion of recuperator to accept treatment is equal to the proportion of susceptible people to refuse drinking alcohol.

The paper is organized as follows: in “Model formulation” section, we set up the model via differential equations. Then we present a global analysis of the model in “Global dynamics of the model” section. In “Control strategy” section, we perform two control strategies. In “Sensitivity analysis and numerical simulations” section, we perform sensitivity analysis and numerical simulations. We finally conclude the paper and give some measures to control alcoholism in “Conclusion and discussions” section.

## Model formulation

In epidemic model, the total population *N* generally is divided into susceptible, represented by *S*, infections, represented by *I* and recovery, represented by *R*. An SIRS model, susceptible people usually infected by infections and become infected individual, after infection acquired immunity, it will be recovery. It allows members of the recovered class to be free of infection and rejoin the susceptible class. Zhu et al. (2012b) introduced an SEIRS epidemic model with the incubation period on complex network. Zhu et al. (2013) proposed a modified epidemic SIS model on an adaptive and weighted contact network, they introduced the general forms of the weight function and presented a new weight called “adaptive weight”. Liu et al. (2013) presented an SIR model with individual’s birth and death on scale-free networks and analyzed the stability of three equilibria. Motivated by these work, we set up a new SIRS alcoholism model on complex network. First, we introduce the individual’s birth and death in our model, Second, we take into account adaptive weight in our model. At last, we studied the effect of alcohol relapse on the spread of the alcoholism. In our model, we divide the whole population into three compartments: the susceptible *S*(*t*), denote the people who do not drink or drink limited; the problem alcoholic *I*(*t*),  denote the people who drink more than daily and weekly limit; the recuperator *R*(*t*),  denote the people who recover from alcoholism after treatment. In order to reflect the heterogeneity of the contact network, it is necessary to consider the node with different degree. Let ${S_k}(t)$, ${I_k}( t )$ and ${R_k}(t)$ denote the densities of susceptible individuals, problem alcoholics and recuperator individuals with degree *k* at time *t* respectively. Then $S( t ) = \sum \nolimits _k P (k){S_k}(t)$, $I(t) = \sum \nolimits _k P (k){I_k}(t )$ and $R(t) = \sum \nolimits _k P (k ){R_k}(t)$ are the average densities of susceptible individuals, problem alcoholics and recuperator individuals respectively, where *P*(*k*) is the probability that a randomly chosen node has degree *k*.

**Fig. 1:**
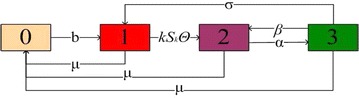
Transfer diagram for alcoholism model

On complex networks, as alcoholism spread in a crowd, every site of *N* is empty or occupied by only one individual. Just as Liu et al. (2013), we give each site a number: 0, 1, 2, 3. We interpret the four states as: state 0: vacant; state 1: a susceptible individual occupied; state 2: a problem alcoholic occupied; state 3: a recuperator individual occupied. The states of the system at time *t* can be described by a set of numbers $\{0, 1, 2, 3\}.$ Each site can change its state at a certain rate. We assume that a birth event occurs at a vacant node at rate *b*. A susceptible individual can be infected through contact with a problem alcoholic. While a problem alcoholic can be cured at rate $\alpha$ or lead to relapse at rate $\beta$ through contact with problem alcoholics or other reasons. All individuals’ death rate is $\mu$ and we assume that alcoholism is not fatal. If a person is dead, the corresponding side becomes vacant. Therefore, the dynamics of ${S_k}\left( t \right)$, ${I_k}\left( t \right)$ and ${R_k}\left( t \right)$ are described by the following differential equations

1 $$\begin{aligned} \left\{ {\begin{array}{l} {\frac{{d{S_k}(t)}}{{dt}} = b( {1 - {S_k}( t ) - {I_k}( t ) - {R_k}( t )} )}\\ \quad \quad \qquad - k{S_k}( t )\Theta ( t ) + \sigma {R_k}(t) - \mu {S_k}( t ),\\ {\frac{{d{I_k}(t)}}{{dt}} = k{S_k}( t )\Theta ( t ) + \beta {R_k}( t ) - \alpha {I_k}(t) - \mu {I_k}(t),}\\ {\frac{{d{R_k}(t)}}{{dt}} = \alpha {I_k}(t) - \beta {R_k}( t ) - \sigma {R_k}(t) - \mu {R_k}(t),} \end{array}} \right. \end{aligned}$$

with initial conditions

2 $$\begin{aligned} \begin{array}{l} {\Omega ^* = \{ \left( {{S_k}(t),{I_k}( t ),{R_k}(t)}\right) \in R_ + ^{3n}|0 \le {S_k}(t) \le 1,\quad 0 \le {I_k}(t) \le 1,\quad 0 \le {R_k}(t) \le 1,}\\ {\quad \qquad k = 1,2, \ldots ,n\} ,} \end{array} \end{aligned}$$

where

3 $${\Theta (t) = \sum \limits _i {{\lambda _{ik}}\frac{{\varphi (i)}}{i}P( {i|k})} {I_i}\left( t \right) ,}$$

and the parameters are all positive constants. The model structure is shown in Fig. 1.

The meanings of the parameters and variables in model (1) and (3) are as follows

- ${b( {1 - {S_k}( t ) - {I_k}( t ) - {R_k}( t )} )}$ represents the new born susceptible individuals per unit time, which is proportional to vacant nodes’ birth rate *b* and the density of vacant nodes ${( {1 - {S_k}( t ) - {I_k}( t ) - {R_k}( t )} )}$.
- ${k{S_k}( t ){\Theta }( t )}$ represents the new problem alcoholic individuals per unit time, which is proportional to the degree *k*, the density of susceptible individuals ${{S_k}( t )}$ and the probability that alcoholism transmits through a link ${{\Theta }( t )}$, while ${\lambda _{ik}}$ is the transmission rate from nodes with degree *i* to nodes with degree *k*, ${\varphi }( i )$ is the infectivity of the problem alcoholic nodes with degree *i*. So $\frac{{\varphi ( i )}}{i}$ is the link’s average infectivity of the problem alcoholic nodes with degree *i*. *P*( *i*|*k* ) is the probability that a node of degree *k* connected to a node of degree *i*. In this paper, we focuses on degree uncorrelated networks. Hence, $P( {i|k} ) = iP(i)/\langle k\rangle$, where $\langle k \rangle = \sum \nolimits _i {iP(i)}$ is the average degree of the network.
- $\mu$ is the natural death rate. Since the disease of alcoholism is assumed not fatal, so there is no disease related death. We assumed that if an individual dies, the corresponding side will become empty. $\alpha$ represents the recovery rate of the problem alcoholics. Some recuperators are likely to recur drinking. The density of relapse alcoholics is $\beta {R_k}$, where $\beta$ means the recurrence rate. $\sigma$ represents the transfer rate from recuperator to susceptible people.

There is little literature about the network model with links’ or nodes’ weights, but the weighted patterns on complex networks have various formats. Weighted patterns are used to represent the different intensities of infection by contact. Usually, the weight between two nodes with degree *i* and *j* are measured by a function of their degrees $\omega ({i,j}) = {\omega _0}{( {ij})^m}$ (Barrat et al. 2004a, b, c; Macdonald et al. 2005), where $\omega _0$ and *m* depend on the specific network. In the *Escherichia coli* metabolic network $m = 0.5;$ in the US airport network $m = 0.8;$ in the scientist collaboration network $m = 0.$ Here, we use a different expression for the weight function $\omega ( {i,j} ) = g(i)g(j)$ (Zhu et al. 2013), where *g*(*k*) is an increasing function of *k*, because the nodes with more connections will be more influential and gain larger weights. Since $\omega ({i,j})$ estimates the links’ weight, the weight of each node ${\Phi _k}$ can also be measured by summing up the weights of links connected to it. Thus, ${\Phi _k} = k\sum \nolimits _i {P({i|k})} \omega ({i,k})$. On uncorrelated networks, ${\Phi _k} = kg(k )\langle {kg(k)} \rangle /\langle k \rangle$. We assume that the node with degree *i* has a fixed transmission rate given by $\lambda i$, and the transmission by the link from the *i*-degree node to a *k*-degree node is measured by the proportion of this link’s weight accounting for the *k*-degree nodes’ weight (Chu et al. 2011). So, we have

4 $${\lambda _{ik}} = \lambda i\frac{{\omega ( {i,k} )}}{{{\Phi _i}}} = \frac{{\lambda g( k )\langle k \rangle }}{{\left\langle {kg( k )} \right\rangle }}$$

In this paper, we also consider people’s health-conscious behavior, so the value of weight function will become less and less as the alcoholism progresses. In particular, if a person has more neighbors, it will be more cautious, therefore the weight will decrease more obviously. Thus, the weight function can be expressed as ${g^{\prime }}( {k,t} ) = g( k )\exp ( { - h( k )I( t )} )$, where *h*(*k*) is an increase function of *k*. The corresponding ${\lambda _{ik}}$ becomes

5 $${\lambda _{ik}^{\prime }} = \frac{{\lambda \langle k \rangle g(k)\exp ( { - h( k)I(t)} )}}{{\left\langle {kg(k)\exp ( { - h( k )I( t)})} \right\rangle }}$$

where $${\langle {kg( k )\exp ( { - h( k )I( t )} )} \rangle = \sum \nolimits _i {ig( i )\exp ( { - h( i )I( t )} )} P( i )}$$.

Substituting (4) and (5) into (3) respectively, we get two ${\Theta }(t)$ respectively

6 $${\Theta ^{\prime }}( t ) = \frac{{\lambda g( k )}}{{\langle {\lambda g( k )} \rangle }}\sum \limits _i {\varphi ( i )} P( i ){I_i}( t ),$$

and

7 $$\Theta ^{\prime \prime }(t) = \frac{{\lambda g(k)\exp ({ - h(k)({ I( t )})} )}}{{\left\langle {\lambda g( k )\exp ({ - h(k)({ I(t)})})} \right\rangle }}\sum \limits _i {{\varphi }(i)} P(i ){I_i}(t)$$

it is clear that when $h(k) = 0$, (7) reduces to (6).

Then, substituting (6) into (1), we obtain the fixed weight system

8 $$\begin{aligned} \left\{ {\begin{array}{l} {\frac{{d{S_k}(t)}}{{dt}} = b\left( {1 - {S_k}\left( t \right) - {I_k}\left( t \right) - {R_k}\left( t \right) } \right) + \sigma {R_k}(t)}\\ \quad \quad \qquad - \mu {S_k}\left( t \right) - \frac{{\lambda g\left( k \right) }}{{\left\langle {kg\left( k \right) } \right\rangle }}k{S_k}\left( t \right) \theta ,\\ {\frac{{d{I_k}(t)}}{{dt}} = \frac{{\lambda g\left( k \right) }}{{\left\langle {kg\left( k \right) } \right\rangle }}k{S_k}\left( t \right) \theta + \beta {R_k}\left( t \right) - \alpha {I_k}(t) - \mu {I_k}(t),}\\ {\frac{{d{R_k}(t)}}{{dt}} = \alpha {I_k}(t) - \beta {R_k}\left( t \right) - \sigma {R_k}(t) - \mu {R_k}(t),} \end{array}} \right. \end{aligned}$$

and substituting (7) into (1), we obtain the adaptive weight system

9 $$\begin{aligned} \left\{ {\begin{array}{l} \begin{array}{l} \frac{{d{S_k}(t)}}{{dt}} = b\left( {1 - {S_k}\left( t \right) - {I_k}\left( t \right) - {R_k}\left( t \right) } \right) \\ \quad \quad \qquad - \frac{{\lambda g\left( k \right) \exp \left( { - h\left( k \right) I\left( t \right) } \right) }}{{\left\langle {kg\left( k \right) \exp \left( { - h\left( k \right) I\left( t \right) } \right) } \right\rangle }}k{S_k}\left( t \right) \theta + \sigma {R_k}(t) - \mu {S_k}\left( t \right) , \end{array}\\ {\frac{{d{I_k}(t)}}{{dt}} = \frac{{\lambda g\left( k \right) \exp \left( { - h\left( k \right) I\left( t \right) } \right) }}{{\left\langle {kg\left( k \right) \exp \left( { - h\left( k \right) I\left( t \right) } \right) } \right\rangle }}k{S_k}\left( t \right) \theta }\\ \quad \quad \qquad +\,\beta {R_k}\left( t \right) - \alpha {I_k}(t) - \mu {I_k}(t),\\ {\frac{{d{R_k}(t)}}{{dt}} = \alpha {I_k}(t) - \beta {R_k}\left( t \right) - \sigma {R_k}(t) - \mu {R_k}(t),} \end{array}} \right. \end{aligned}$$

where ${{\theta }(t) = \sum \nolimits _i {{\varphi }} ( i){I_i}(t)P(i)}$ in (8) and (9).

Let ${N_k}( t ) = {S_k}( t ) + {I_k}( t ) + {R_k}( t )$ be the density of the whole individuals with degree *k*, $k = 1,2, \ldots ,n$. Then adding the three equations in (8) or (9) gives

10 $$\frac{{d{N_k}( t )}}{{dt}} = b - ( {b +\mu } ){N_k}( t ).$$

By Eq. (10), we get that ${N_k}( t) = \frac{b}{{b + \mu }} + {N_k}(0){e^{ - ( {b + \mu } )t}}$, where ${N_k}(0)$ represents the initial density of whole population with degree *k*. Hence, $\mathop {\lim }\nolimits _{t \rightarrow \infty } \sup {N_k}(t) = \frac{b}{{b + \mu }}$, then ${N_k}(t) = {S_k}(t) + {I_k}(t) + {R_k}(t) \le \frac{b}{{b + \mu }}$ for all $t \ge 0$.

Due to the limit system and original system have the same dynamic behaviors for a long time. And ${S_k}( t ) = \frac{b}{{b + \mu }} - {A_k}( t ) - {I_k}( t ) - {R_k}( t )$ at steady-state, it is sufficient to study the limiting systems

11 $$\begin{aligned} \left\{ {\begin{array}{l} {\frac{{d{I_k}(t)}}{{dt}} = \frac{{\lambda kg\left( k \right) }}{{\left\langle {kg\left( k \right) } \right\rangle }}\left( {\frac{b}{{b + \mu }} - {I_k}\left( t \right) - {R_k}\left( t \right) } \right) \theta \left( t \right) }\\ \quad \quad \qquad +\, \beta {R_k}(t) - \alpha {I_k}(t) - \mu {I_k}(t),\\ {\frac{{d{R_k}(t)}}{{dt}} = \alpha {I_k}(t) - \beta {R_k}(t) - \sigma {R_k}(t) - \mu {R_k}(t),} \end{array}} \right. \end{aligned}$$

and

12 $$\begin{aligned} \left\{ {\begin{array}{l} \begin{array}{l} \frac{{d{I_k}(t)}}{{dt}} = \frac{{\lambda g( k)\exp ({ - h( k)I( t)} )}}{{\left\langle {kg(k)\exp ({ - h(k )I(t)})} \right\rangle }}\left( {\frac{b}{{b + \mu }} - {I_k}(t) - {R_k}(t )} \right) \theta (t)\\ \quad \quad \qquad +\, \beta {R_k}(t)- \alpha {I_k}(t) - \mu {I_k}(t), \end{array}\\ {\frac{{d{R_k}(t)}}{{dt}} = \alpha {I_k}(t) - \beta {R_k}(t) - \sigma {R_k}(t) - \mu {R_k}(t),} \end{array}} \right. \end{aligned}$$

It is easy to obtain that ${0 \le {I_k}(t) \le \frac{b}{{b + \mu }}}$ and ${0 \le {R_k}(t) \le \frac{b}{{b + \mu }}}$ for $t\ge 0$. So the region $\Omega = \{ {({{I_k},{R_k}})|0 \le {I_k}(t) \le \frac{b}{{b + \mu }},0 \le {R_k}(t) \le \frac{b}{{b + \mu }},k = 1,2,\ldots ,n}\}$ is the positive invariant for both (11) and (12).

## Global dynamics of the model

### The basic reproduction number *R*_0_

Here, we first calculate the fix weight model’s basic reproduction number. Using the next generation method in Driessche and Watmough (2002), it is clear that model (11) has an alcohol free equilibrium ${E_0} = {({0,0, \ldots ,0})_{2k}}$. System (11) can be written as

$$\frac{{dx}}{{dt}} = {\mathcal{F}}(x)-{\mathcal{V}}( x ),$$

and

$$x = {({{I_k},{R_k}})^T},$$

where the rate of appearance of new infections is

$$\begin{aligned} {{\mathcal{F}}}(x) = \left( {\begin{array}{c} {\frac{{\lambda kg(k)\theta }}{{\langle {kg( k)}\rangle }}\left( {\frac{b}{{b + \mu }} - {I_k} - {R_k}} \right) }\\ 0 \end{array}} \right) , \end{aligned}$$

and the rate of transfer of individuals out of compartments is

$$\begin{aligned} {{\mathcal{V}}}(x) = \left( {\begin{array}{l} { - \beta {R_k} +( {\alpha + \mu }){I_k}}\\ { - \alpha {I_k} +( {\beta + \sigma + \mu }){R_k}} \end{array}} \right) . \end{aligned}$$

The Jacobian matrices of ${\mathcal{F}}( x)$ and ${\mathcal{V}}(x)$ at the alcohol free equilibrium ${E_0}$ are

13 $$\begin{aligned} F= {} DF({{E_0}}) = \left( {\begin{array}{cc} {{F_{11}}}&{}\quad 0\\ 0&{}\quad 0 \end{array}} \right) \end{aligned}$$

14 $$\begin{aligned} V= {} DV\left( {{E_0}} \right) = \left( {\begin{array}{cc} {\left( {\alpha + \mu } \right) E}&{}\quad { - \beta E}\\ { - \alpha E}&{}\quad {({\beta + \sigma + \mu })E} \end{array}} \right) , \end{aligned}$$

where

$$\begin{aligned}&{F_{11}}\\&\quad = \frac{{\lambda b}}{{\left( {b + \mu } \right) \left\langle {kg\left( k \right) } \right\rangle }}\left( {\begin{array}{cccc} {g\left( 1 \right) \varphi \left( 1 \right) P\left( 1 \right) }&{}\quad {g\left( 1 \right) \varphi \left( 2 \right) P\left( 2 \right) }&{}\quad \cdots &{}\quad {g\left( 1 \right) \varphi \left( n \right) P\left( n \right) }\\ {2g\left( 2 \right) \varphi \left( 1 \right) P\left( 1 \right) }&{}\quad {2g\left( 2 \right) \varphi \left( 2 \right) P\left( 2 \right) }&{}\quad \cdots &{}\quad {2g\left( 2 \right) \varphi \left( n \right) P\left( n \right) }\\ \vdots &{}\quad \vdots &{}\quad \ddots &{}\quad \vdots \\ {ng\left( n \right) \varphi \left( 1 \right) P\left( 1 \right) }&{}\quad {ng\left( n \right) \varphi \left( 2 \right) P\left( 2 \right) }&{}\quad \cdots &{}\quad {ng\left( n \right) \varphi \left( n \right) P\left( n \right) } \end{array}} \right) , \end{aligned}$$

and *E* is identity matrix, 0 is zero matrix. It is clear that *V* is a nonsingular M-matrix and *F* is a nonnegative matrix. According to the concept of next generation matrix and reproduction number given in Driessche and Watmough (2002), the reproduction number of (11) equals to

15 $${R_0} = \rho \left( {F{V^{ - 1}}} \right) = \frac{{\lambda b( {\beta + \sigma + \mu })\left\langle {kg( k )\varphi (k)}\right\rangle }}{{({b + \mu } )({\alpha ( {\sigma + \mu }) + \mu ( {\beta + \sigma + \mu })} )\left\langle {kg(k )} \right\rangle }},$$

where $$\langle {kg( k )\varphi ( k )} \rangle = \sum \nolimits _{i = 1}^k {i\varphi ( i )g( i )P( i )}$$.

Coincidentally, we get that matrices *F* and *V* in model (12) are the same as that in model (11). Therefore, the reproduction number ${R_0}$ of model (12) is also given by (15), which implies that the adaptive weights cannot change the propagation threshold.

According to the above process and Theorem 2 in Driessche and Watmough (2002), we obtain the following results.

#### **Theorem 1**

*For the two alcoholism models* (11) *and* (12), *we have*

1. *Both of their basic reproductive number are equal to*${R_0}$*in* (15).
2. *If*${R_0} < 1$, *the alcohol free equilibrium*${E_0}$*of* (11) *and* (12) *is locally asymptotically stable, but unstable if*${R_0} > 1$, *where*${R_0}$*is defined by* (15).

*Next, we will investigate the global stability of the alcohol free equilibrium and the globally attractive of the alcoholism equilibrium of model* (11). *Since the stability of the equilibria in model* (12) *are difficult to demonstrate, so we only give some numerical simulations to discuss it at* “Sensitivity analysis and numerical simulations” section.

### Uniqueness of the alcoholism equilibrium

We first give the following Lemma which guarantee that the density of population in each compartment cannot become negative or greater than ${\frac{b}{{b + \mu }}}$.

Let ${I_1} = {y_1},{I_2} = {y_2}, \ldots ,{I_n} = {y_n},{R_1} = {y_{n + 1}},{R_2} = {y_{n + 2}}, \ldots ,{R_n} = {y_{2n}}$, we study the system for $({y_1}, \ldots ,{y_n},{y_{n + 1}}, \ldots ,{y_{2n}}) \in \Omega = \prod \nolimits _{i = 1}^{2n} {\left[ {0,\frac{b}{{b + \mu }}} \right] }$.

#### **Lemma 1**

*The set*$\Omega$*is the positively invariant for system* (11).

#### *Proof*

We will show that if $y(0) \in \Omega$, then $y( t) \in \Omega$ for all $t > 0$. Denote

$$\begin{aligned} \partial {\Omega _1}&= {} \left\{ {y \in \Omega |{y_i} = 0\quad for\;some\;i} \right\} ,\\ \partial {\Omega _2}&= {} \left\{ {y \in \Omega |{y_i} = \frac{b}{{b + \mu }}\quad for\;some\;i} \right\} , \end{aligned}$$

where $i = 1,2, \ldots ,2n$. Let the ‘outer normals’ be denoted by $\xi _i^1 = {(\underbrace{0, \ldots , - 1}_{ith}, \ldots ,0)_{2n}}$ and $\xi _i^2 = {(\underbrace{0, \ldots , + 1}_{ith}, \ldots ,0)_{2n}}$. We use the Nagumo’s result in Yorke (1967). Since $\Omega$ is a 2*n*-dimensional rectangle. From (11), it is easy to obtain that for $i=1,2, \ldots ,n$.

$$\begin{aligned}&\left( \left. \frac{{dy}}{{dt}}\right| _{{y_i} = 0} \cdot \xi _i^1 \right) = - \beta {y_{n + i}} \le 0,\\&\left( \left. \frac{{dy}}{{dt}}\right| _{{y_{n + i}} = 0} \cdot \xi _{n + i}^1 \right) = - \alpha {y_i} \le 0,\\&\left( \left. \frac{{dy}}{{dt}}\right| _{{y_i} = \frac{b}{{b + \mu }}} \cdot \xi _i^2 \right) = - ({\alpha + \mu })\frac{b}{{b + \mu }} \le 0,\\&\left( \left. \frac{{dy}}{{dt}}\right| _{{y_{n + i}} = \frac{b}{{b + \mu }}} \cdot \xi _{n + i}^2 \right) = - ( {\beta + \sigma + \mu } )\frac{b}{{b + \mu }} \le 0. \end{aligned}$$

So, any solution that starts in $y \in \partial {\Omega _1} \cup \partial {\Omega _2}$ stays inside ${\Omega }$.

Furthermore, we will ascertain the uniqueness of the alcoholism equilibrium. We give the following theorem.

#### **Theorem 2**

*There exists a unique alcoholism equilibrium*${y^*} = ( {y_1^*,\ldots ,y_n^*,y_{n + 1}^*, \ldots ,y_{2n}^*})$*in model* (11) *when*${R_0} > 1$.

#### *Proof*

The alcoholism equilibrium ${y^*} = ( {y_1^*,\ldots ,y_n^*,y_{n + 1}^*, \ldots ,y_{2n}^*})$ of system (11) is determined by equations

$$\begin{aligned} \left\{ \begin{array}{l} \frac{{\lambda ig\left( i \right) \theta }}{{\left\langle {kg\left( k \right) } \right\rangle }}\left( {\frac{b}{{b + \mu }} - y_i^* - y_{n + i}^*} \right) + \beta y_{n + i}^* - \left( {\alpha + \mu } \right) y_i^* = 0,\\ \alpha y_i^* - \beta y_{n + i}^* - \sigma y_{n + i}^* - \mu y_{n + i}^* = 0, \end{array} \right. \end{aligned}$$

a direct calculation yields

$$\begin{aligned}&{y_i} = \frac{{b\lambda ig\left( i \right) \left( {\beta + \sigma + \mu } \right) \theta }}{{\left( {b + \mu } \right) \left[ {\left( {\alpha + \mu + i\theta } \right) \left( {\beta + \sigma + \mu } \right) + \alpha i\theta - \alpha \beta } \right] \left\langle {kg\left( k \right) } \right\rangle }}\\&{y_{n + i}} = \frac{{\alpha b\lambda ig\left( i \right) \theta }}{{\left( {b + \mu } \right) \left[ {\left( {\alpha + \mu + i\theta } \right) \left( {\beta + \sigma + \mu } \right) + \alpha i\theta - \alpha \beta } \right] \left\langle {kg\left( k \right) } \right\rangle }} \end{aligned}$$

Then we get a self-consistency equation of $\theta$ as follows

$$\begin{aligned} \theta&= {} \sum \limits _i {\varphi \left( i \right) } P\left( i \right) {y_i} \\&= {} \sum \limits _i {\varphi \left( i \right) } P\left( i \right) \frac{{b\lambda ig\left( i \right) \left( {\beta + \sigma + \mu } \right) \theta }}{{\left( {b + \mu } \right) \left[ {\left( {\alpha + \mu + i\theta } \right) \left( {\beta + \sigma + \mu } \right) + \alpha i\theta - \alpha \beta } \right] \left\langle {kg\left( k \right) } \right\rangle }} \end{aligned}$$

Obviously, ${\theta } = 0$ satisfies above equation, then ${y_i} = {y_{n + i}} = 0$, which is the alcohol free equilibrium of (11). We transform the self-consistency equation form as $\theta f(\theta ) = 0$, where

$$\begin{aligned}&f(\theta )\\&\quad = 1 - \sum \limits _i {\varphi (i )} P(i)\frac{{b\lambda ig(i)( {\beta + \sigma + \mu } )}}{{({b + \mu })\left[ {\left( {\alpha + \mu + i\theta } \right) \left( {\beta + \sigma + \mu } \right) + \alpha i\theta - \alpha \beta } \right] \left\langle {kg\left( k \right) } \right\rangle }} \end{aligned}$$

since

$$\begin{aligned}&{f^{\prime }}\left( \theta \right) \\&\quad = \sum \limits _i {\varphi \left( i \right) } P\left( i \right) \frac{{b\lambda {i^2}g\left( i \right) \left( {\beta + \sigma + \mu } \right) \left( {\beta + \sigma + \alpha + \mu } \right) }}{{\left( {b + \mu } \right) \left\langle {kg\left( k \right) } \right\rangle {{\left[ {\left( {\alpha + \mu + i\theta } \right) \left( {\beta + \sigma + \mu } \right) + \alpha i\theta - \alpha \beta } \right] }^2}}} > 0 \end{aligned}$$

and

$$\mathop {\lim }\limits _{\theta \rightarrow \infty } f\left( \theta \right) = 1$$

the equation $f( \theta) = 0$ has a unique non-trivial solution ${\theta ^*}$ if and only if $f( 0) < 0.$

$$\begin{aligned} f\left( 0 \right)&= {} 1 - \frac{{\sum \nolimits _i {\varphi \left( i \right) } P\left( i \right) ig\left( i \right) b\lambda \left( {\beta + \sigma + \mu } \right) }}{{\left( {b + \mu } \right) \left[ {\left( {\alpha + \mu } \right) \left( {\beta + \sigma + \mu } \right) - \alpha \beta } \right] \left\langle {kg\left( k \right) } \right\rangle }}\\&= {} 1 - \frac{{b\lambda \left( {\beta + \sigma + \mu } \right) \left\langle {kg\left( k \right) \varphi \left( k \right) } \right\rangle }}{{\left( {b + \mu } \right) \left[ {\left( {\alpha + \mu } \right) \left( {\beta + \sigma + \mu } \right) - \alpha \beta } \right] \left\langle {kg\left( k \right) } \right\rangle }}\\&= {} 1 - \frac{{b\lambda \left( {\beta + \sigma + \mu } \right) \left\langle {kg\left( k \right) \varphi \left( k \right) } \right\rangle }}{{\left( {b + \mu } \right) \left[ {\alpha \left( {\sigma + \mu } \right) + \mu \left( {\beta + \sigma + \mu } \right) } \right] \left\langle {kg\left( k \right) } \right\rangle }}< 0 \end{aligned}$$

then ${R_0} = \frac{{b\lambda ( {\beta + \sigma + \mu } )\langle {kg( k )\varphi ( k )} \rangle }}{{( {b + \mu } )[ {\alpha ( {\sigma + \mu } ) + \mu ( {\beta + \sigma + \mu } )} ]\langle {kg( k )} \rangle }} > 1$, through which the alcoholism equilibrium is admitted. The proof is completed.

### Global stability of the alcohol free equilibrium

Here, we use the method in Lajmanovich and Yorke (1976), d’Onofrio (2008) to demonstrate the global behavior of the system (11). By letting $y = {({y_1}, \ldots ,{y_n},{y_{n + 1}}, \ldots ,{y_{2n}})^T}$. Then, equations in (11) can be rewritten as a form

16 $$\frac{{dy}}{{dt}} = Ay + H(y),$$

where *Ay* is the linear part, *H*( *y*) is the nonlinear part and

$$\begin{aligned} A&= {} \left( {\begin{array}{cc} {{A_{11}}}&{}\quad {\beta E}\\ {\alpha E}&{}\quad { - \left( {\beta + \sigma + \mu } \right) E} \end{array}} \right) ,\\ N(y)&= {} - {{\left( {\begin{array}{cccccc} {\frac{{\lambda g\left( 1 \right) \left( {{y_1} + {y_{n + 1}}} \right) \theta }}{{\left\langle {kg\left( k \right) } \right\rangle }}}&\quad \ldots&\quad {\frac{{n\lambda g\left( n \right) \left( {{y_n} + {y_{2n}}} \right) \theta }}{{\left\langle {kg\left( k \right) } \right\rangle }}}&\quad 0&\quad \ldots&\quad 0 \end{array}} \right) }^T}, \end{aligned}$$

where *E* is unit matrix and

$$\begin{aligned}&{A_{11}}\\&\quad = \left( {\begin{array}{cccc} {\frac{{\lambda bg\left( 1 \right) \varphi \left( 1 \right) P\left( 1 \right) }}{{\left( {b + \mu } \right) \left\langle {kg\left( k \right) } \right\rangle }} - \left( {\alpha + \mu } \right) }&{}\quad {\frac{{\lambda bg\left( 1 \right) \varphi \left( 2 \right) P\left( 2 \right) }}{{\left( {b + \mu } \right) \left\langle {kg\left( k \right) } \right\rangle }}}&{}\quad \ldots &{}\quad {\frac{{\lambda bg\left( 1 \right) \varphi \left( n \right) P\left( n \right) }}{{\left( {b + \mu } \right) \left\langle {kg\left( k \right) } \right\rangle }}}\\ {\frac{{2\lambda bg\left( 2 \right) \varphi \left( 1 \right) P\left( 1 \right) }}{{\left( {b + \mu } \right) \left\langle {kg\left( k \right) } \right\rangle }}}&{}\quad {\frac{{2\lambda bg\left( 2 \right) \varphi \left( 2 \right) P\left( 2 \right) }}{{\left( {b + \mu } \right) \left\langle {kg\left( k \right) } \right\rangle }} - \left( {\alpha + \mu } \right) }&{}\quad \ldots &{}\quad {\frac{{2\lambda bg\left( 2 \right) \varphi \left( n \right) P\left( n \right) }}{{\left( {b + \mu } \right) \left\langle {kg\left( k \right) } \right\rangle }}}\\ \vdots &{}\quad \vdots &{}\quad \ddots &{}\quad \vdots \\ {\frac{{n\lambda bg\left( n \right) \varphi \left( 1 \right) P\left( 1 \right) }}{{\left( {b + \mu } \right) \left\langle {kg\left( k \right) } \right\rangle }}}&{}\quad {\frac{{n\lambda bg\left( n \right) \varphi \left( 2 \right) P\left( 2 \right) }}{{\left( {b + \mu } \right) \left\langle {kg\left( k \right) } \right\rangle }}}&{}\quad \ldots &{}\quad {\frac{{n\lambda bg\left( n \right) \varphi \left( n \right) P\left( n \right) }}{{\left( {b + \mu } \right) \left\langle {kg\left( k \right) } \right\rangle }} - \left( {\alpha + \mu } \right) } \end{array}} \right) \end{aligned}$$

#### **Lemma 2**

*Let*$A = {( {{a_{ij}}})_{n \times n}}$*be an*$n \times n$*matrix, and assume*${a_{ij}} \ge 0$*whenever*$i \ne j$. *Then there exists an eigenvector*$\omega$*ofAsuch that*$\omega \ge 0$, *and the corresponding eigenvalue isS*(*A*) .

*The stability modulusS*(*A*) *is defined by*$S( A ) = \max {\mathrm{Re}} {\lambda _i}$, $i = 1, \ldots ,n$, *where*${\lambda _i}$*are the eigenvalues ofA*.

#### *Proof*

Choose $c \in R$, such that $c + {a_{ii}} \ge 0$, for $i = 1,2, \ldots , n$. Then $A + cE$ is an $n \times n$ nonnegative matrix. Therefore, by Theorem 2.20 from Varga (2000), there exists a nonnegative eigenvector $\omega \ge 0$ with nonnegative real eigenvalue equal to its spectral radius $\rho ( {A + cE} )$. So we have $({A + cE})\omega = \rho ( {A + cE})\omega$, where *E* is the unit matrix. Then, $A\omega = ( {\rho ( {A + cE} ) - c} )\omega$, so $\omega$ is also an eigenvector of *A*, and the corresponding eigenvalue is $( {\rho ( {A + cE} ) - c} )$. If $\lambda$ is any eigenvalue of *A*, then $\lambda + c$ is an eigenvalue of ${A + cE}$, so $| {\lambda + c} | \le \rho ( {A + cE} )$, then we have $\lambda + c \le \rho ( {A + cE} )$, and $\lambda \le \rho ( {A + cE} ) - c$, therefore ${\mathrm{Re}} \lambda \le \rho ( {A + cE} ) - c$, that is to say $\rho ( {A + cE} ) - c$ is the maximum real part of all eigenvalues of A, so $S( A ) = \rho ( {A + cE} ) - c$. The proof is completed.

#### **Lemma 3**

(Lajmanovich and Yorke 1976) *Consider the system*

17 $$\frac{{dy}}{{dt}} = Ay + N(y),$$

*whereAis an*$n \times n$*matrix andN*(*y*) *is continuously differentiable in a region*$D \subset {R^n}$. *Assume*

(i) *the compact convex set*$C \subset D$*is positively invariant with respect to the system* (17), *and*$0 \in C$;
(ii) $$\mathop {\lim }\nolimits _{y \rightarrow 0} \Vert {N( y )} \Vert /\Vert y \Vert = 0$$;
(iii) *there exist*$r > 0$*and a (real) eigenvector*$\omega$*of*${A^T}$*such that*$(\omega \cdot y)\ge r\Vert y \Vert$*for all*$y \in C$;
(iv) $( {\omega \cdot N(y)}) \le 0$*for all*$y \in C$;
(v) $y=0$*is the largest positively invariant set contained in*$H = \{ {{y \in C }|( {\omega \cdot N( y )} ) = 0} \}$.

*Then either*${y = 0}$*is globally asymptotically stable inC*, *or for any*${y_0} \in C - \{ 0 \}$*the solution*$\phi ( {t,{y_0}} )$*of* (17) *satisfies*$\mathop {\lim }\nolimits _{t \rightarrow \infty } \inf \Vert {\phi ( {t,{y_0}} )} \Vert \ge m$, *independent of*${y_0}$. *Moreover, there exists a constant solution of* (17), $y = k, k \in C - \{ 0 \}$.

#### **Theorem 3**

*For system* (11). *When*${R_0} < 1$, *there exists an alcohol free equilibrium*$y = 0$*is globally asymptotically stable in*$\Omega$. *When*${R_0} > 1$, *there exists an alcoholism equilibrium*${y^*}$*is permanent in*$\Omega - \{ 0 \}$, *that is to say, there exists anmsatisfies*$\mathop {\lim }\nolimits _{t \rightarrow \infty } \inf \Vert {{y^*}} \Vert \ge m$.

#### *Proof*

We will confirm that the system (11) satisfies all the hypotheses of Lemma 3.

- Condition (i): Lemma 3 is satisfied if we suppose that $C = \Omega \subset {R^{2n}}$.
- Condition (ii): using the mean inequality and limit rule can validate the conclusion.
- Condition (iii): notice that $A^T$ is an ${2n \times 2n}$ matrix with ${a_{ij}} \ge 0$ whenever $i \ne j$, then from Lemma 2, there exists an eigenvector $\omega = ( {{\omega _1},{\omega _{2,}} \ldots ,{\omega _{2n}}}) \ge 0$ of $A^T$ and the associated eigenvalue is $S( {A^T})$. Let $r = \mathop {\min }\nolimits _{1 \le i \le 2n} {\omega _i} > 0$, for $y \in {\Omega }$, $( {\omega \cdot y} ) \ge r\sqrt{\sum \nolimits _{i = 1}^{2n} {y_i^2} }$, therefore $( {\omega \cdot y}) \ge r||y||$ for all $y \in {\Omega }$.
- Condition (iv): we know $\omega > 0$ and $N( y) \le 0$, so it is clearly satisfied.
- Condition (v): let $H = \{ {{y \in {\Omega }}|( {\omega \cdot N(y)}) = 0} \}$. If ${y \in {H} }$, then ${\sum \nolimits _i {\frac{{i\lambda {\omega _i}g( i )( {{y_i} + {y_{2i}}} )\theta }}{{\langle {kg( k )} \rangle }} = 0} }$ for $i=1,2,\ldots ,2n$. But since each term of the sum is nonnegative, then we get that $\frac{{i\lambda {\omega _i}g( i )( {{y_i} + {y_{2i}}} )\sum \nolimits _j {\varphi ( j )P( j ){y_j}} }}{{\langle {kg( k )} \rangle }} = 0$. If this equation is established, then $y_j=0$ or ${y_i} = {y_{n + i}} = 0$. From system (11), if $y_j=0$ then ${y_{n + j}} = 0$ for $j = 1,2, \ldots , n$. That is $( {{y_1}, \ldots ,{y_n},{y_{n + 1}}, \ldots ,{y_{2n}}} ) = 0$. So, the only invariant set respect to (11) contained in *H* is $y=0$, condition (v) is satisfied. This completes the proof.

### Globally attractive of the alcoholism equilibrium

#### **Theorem 4**

*When*${R_0} > 1$, *the only alcoholism equilibrium*$y = {y^*}$*in model* (11) *is globally attractive in*$\Omega - \{ 0 \}$.

#### *Proof*

We define the following functions, $M:\Omega \rightarrow R$ and $m:\Omega \rightarrow R$ for $y \in \Omega$, where $M( y ) = {\max _i}( {\frac{{{y_i}}}{{y_i^*}}} )$, $m( y ) = {\min _i}( {\frac{{{y_i}}}{{y_i^*}}} )$ are continuous and the right-hand derivative exists along solutions of (11). Let ${y = y( t )}$ be a solution of (11), we may assume that $M( {y( t )} ) = \frac{{{y_{{i_0}}}( t )}}{{y_{{i_0}}^*( t )}}$, ${i_0} = 1,2, \ldots ,2n$ and $t \in [ {{t_0},{t_0} + \varepsilon } ]$. For a given $t_0$ and for sufficiently small $\varepsilon > 0$

$$\left.{M^{\prime }}\right|_{({2.11})}( {y( {{t_0}} )} ) =\frac{{{y_{{i_0}}^{\prime }}( {{t_0}} )}}{{y_{{i_0}}^*}}, \quad for\;t \in [ {{t_0},{t_0} + \varepsilon } ].$$

from (11), if $1 \le {i_0} \le n$, we have

$$\begin{aligned} y_{{i_0}}^*\frac{{y_{{i_0}}^{\prime }\left( {{t_0}} \right) }}{{{y_{{i_0}}}\left( {{t_0}} \right) }}&= {} \frac{{\lambda {i_0}g\left( {{i_0}} \right) \theta }}{{\left\langle {kg\left( k \right) } \right\rangle }}\left( {\frac{b}{{b + \mu }} - {y_{{i_0}}}\left( {{t_0}} \right) - {y_{n + {i_0}}}\left( {{t_0}} \right) } \right) \frac{{y_{{i_0}}^*}}{{{y_{{i_0}}}\left( {{t_0}} \right) }}\\&\quad + \beta {y_{n + {i_0}}}\left( {{t_0}} \right) \frac{{y_{{i_0}}^*}}{{{y_{{i_0}}}\left( {{t_0}} \right) }} - \left( {\alpha + \mu } \right) y_{{i_0}}^*, \end{aligned}$$

or for $i = 1,2, \ldots ,n$ and ${i_0} = n + i$, we have

$$y_{n + i}^*\frac{{y_{n + i}^{\prime }\left( {{t_0}} \right) }}{{{y_{n + i}}\left( {{t_0}} \right) }} = \alpha {y_i}\left( {{t_0}} \right) \frac{{y_{n + i}^*}}{{{y_{n + i}}\left( {{t_0}} \right) }} - \beta y_{n + i}^* - \sigma y_{n + i}^* - \mu y_{n + i}^*.$$

According to the definition of *M*( *y*( *t*) ), we know

$$\frac{{{y_{{i_0}}}( {{t_0}} )}}{{y_{{i_0}}^*}} \ge \frac{{{y_i}( {{t_0}} )}}{{y_i^*}},\quad i = 1,2, \ldots ,2n.$$

Then, if $M( {y( {{t_0}} )} ) > 1$, for $1 \le {i_0} \le n$ we have

$$\begin{aligned}&y_{{i_0}}^*\frac{{y_{{i_0}}^{\prime }\left( {{t_0}} \right) }}{{{y_{{i_0}}}\left( {{t_0}} \right) }} < \frac{{\lambda {i_0}g\left( {{i_0}} \right) \theta \left( {y_{{i_0}}^*} \right) }}{{\left\langle {kg\left( k \right) } \right\rangle }}\left( {\frac{b}{{b + \mu }} - y_{{i_0}}^*\left( {{t_0}} \right) - y_{n + {i_0}}^*\left( {{t_0}} \right) } \right) \\&\quad \quad \qquad + \beta y_{n + {i_0}}^*\left( {{t_0}} \right) - \left( {\alpha + \mu } \right) y_{{i_0}}^*=0, \end{aligned}$$

or for $i = 1,2, \ldots ,n$ and ${i_0} = n + i$

$$y_{n + i}^*\frac{{y_{n + i}^{\prime }\left( {{t_0}} \right) }}{{{y_{n + i}}\left( {{t_0}} \right) }} < \alpha {y_i}\left( {{t_0}} \right) \frac{{y_{n + i}^*}}{{{y_{n + i}}\left( {{t_0}} \right) }} - \beta y_{n + i}^* - \sigma y_{n + i}^* - \mu y_{n + i}^*=0,$$

and since ${y_{{i_0}}^* > 0}$ and ${{y_{{i_0}}}( {{t_0}} ) > 0}$, we conclude that ${{y_{{i_0}}^{\prime }}( {{t_0}} ) < 0}$. Therefore, if $M( {y( {{t_0}} )} ) > 1$, then ${M^{\prime }}{|_{( 2.11 )}}( {y( {{t_0}} )} ) < 0$. Similarly, we can testify that if $M( {y( {{t_0}} )} ) = 1$, ${{y_{{i_0}}^{\prime }}( {{t_0}} ) \le 0}$. And if $m( {y( {{t_0}} )} ) < 1$, then ${m^{\prime }}{|_{( 2.11 )}}( {y( {{t_0}} )} ) > 0$. If $m( {y( {{t_0}} )} ) = 1$, then ${m^{\prime }}{|_{( 2.11 )}}( {y( {{t_0}} )} ) \le 0$. Denote

$$\begin{aligned} Q(y)&= {} \max \left\{ {M( y) - 1,0} \right\} ,\\ q( y)&= {} \max \left\{ {1 - m( y ),0} \right\} . \end{aligned}$$

Both *Q*( *y* ) and *q*( *y* ) are continuous and non-negative for $y \in \Omega$. Notice that

$$\begin{aligned} Q{|_{(2.11)}}( {y( t )} )&\le {} 0,\\ q{|_{(2.11)}}( {y( t )} )&\le {} 0. \end{aligned}$$

Let ${H_Q} = \{ {y \in \Omega |{Q^{\prime }}{|_{( {2.11})}}( {y(t )}) = 0}\}$ and ${H_q} = \{ {y \in \Omega |{q^{\prime }}{|_{( {2.11} )}}({y(t )}) = 0} \}$, then ${H_Q} = \{ {y|0 \le {y_i} \le y_i^*} \}$ and ${H_q} = \{ {y|y_i^* \le {y_i} \le \frac{b}{{b + \mu }}} \} \cup \{ 0 \}$. According to the LaSalle invariant set principle, any solution of (11) starting in $\Omega$ will approach ${H_Q} \cap {H_q} = \{ {{y^*}} \} \cup \{ 0 \}$. But if ${y(t) \ne 0}$, by Theorem 3 we know that $\mathop {\lim }\nolimits _{t \rightarrow \infty } \inf \Vert {y(t)} \Vert \ge A > 0$. Then we conclude that any solution *y*(*t*) of (11), such that $y( 0 ) \in \Omega - \{ 0 \}$, satisfies $\mathop {\lim }\nolimits _{t \rightarrow \infty } y(t ) = {y^*}$, so $y = {y^*}$ is globally attractive in $\Omega - \{ 0 \}$.

## Control strategy

Timely stopping recuperator recurrence drinking alcohol and stopping the susceptible people to drink are two important and effective ways to prevent the spread of the alcoholism. Next, we give two kinds of control strategies for the treatment of recuperator for stopping relapsing and preventing the susceptible people to drink, respectively. Due to the fixed weight model and adaptive weight model have the same basic reproductive number, so we only study the control strategies of the fixed weight model.

### Proportion treatment

Let $\varepsilon$ be the treatment proportion for recuperator, $0< \varepsilon < 1$, then system (11) becomes

18 $$\begin{aligned} \left\{ {\begin{array}{l} \begin{array}{l} \frac{{d{I_k}(t)}}{{dt}} = \frac{{\lambda kg\left( k \right) }}{{\left\langle {kg\left( k \right) } \right\rangle }}\left( {\frac{b}{{b + \mu }} - {I_k}\left( t \right) - {R_k}\left( t \right) } \right) \theta \left( t \right) \\ \quad \quad \qquad - \left( {\alpha + \mu } \right) {I_k}(t) + \beta \left( {1 - \varepsilon } \right) {R_k}(t), \end{array}\\ {\frac{{d{R_k}(t)}}{{dt}} = \alpha {I_k}(t) - \beta \left( {1 - \varepsilon } \right) {R_k}(t) - \sigma {R_k}(t) - \mu {R_k}(t),} \end{array}} \right. \end{aligned}$$

By the next generation method, the basic reproductive number of (18) is

$$\begin{aligned} {{\tilde{R}}_0} = \frac{{b\lambda \left( {\beta \left( {1 - \varepsilon } \right) + \sigma + \mu } \right) \left\langle {kg\left( k \right) \varphi \left( k \right) } \right\rangle }}{{\left( {b + \mu } \right) \left[ {\alpha \left( {\sigma + \mu } \right) + \mu \left( {\beta \left( {1 - \varepsilon } \right) + \sigma + \mu } \right) } \right] \left\langle {kg\left( k \right) } \right\rangle }} \end{aligned}$$

we can find that with the increase of the treatment proportion, ${{\tilde{R}}_0}$ is smaller.

Change the form of ${{\tilde{R}}_0}$, we get that

$$\begin{aligned} {{\tilde{R}}_0}&= {} \frac{{b\lambda \left( {\beta \left( {1 - \varepsilon } \right) + \sigma + \mu } \right) \left\langle {kg\left( k \right) \varphi \left( k \right) } \right\rangle }}{{\left( {b + \mu } \right) \left[ {\alpha \left( {\sigma + \mu } \right) + \mu \left( {\beta \left( {1 - \varepsilon } \right) + \sigma + \mu } \right) } \right] \left\langle {kg\left( k \right) } \right\rangle }}\\&= {} \frac{{b\lambda \left\langle {kg\left( k \right) \varphi \left( k \right) } \right\rangle }}{{\left( {b + \mu } \right) \left[ {\frac{{\alpha \left( {\sigma + \mu } \right) }}{{\left( {\beta \left( {1 - \varepsilon } \right) + \sigma + \mu } \right) }} + \mu } \right] \left\langle {kg\left( k \right) } \right\rangle }}\\&< {} \frac{{b\lambda \left\langle {kg\left( k \right) \varphi \left( k \right) } \right\rangle }}{{\left( {b + \mu } \right) \left[ {\frac{{\alpha \left( {\sigma + \mu } \right) }}{{\left( {\beta + \sigma + \mu } \right) }} + \mu } \right] \left\langle {kg\left( k \right) } \right\rangle }}\\&= {} \frac{{b\lambda \left( {\beta + \sigma + \mu } \right) \left\langle {kg\left( k \right) \varphi \left( k \right) } \right\rangle }}{{\left( {b + \mu } \right) \left[ {\alpha \left( {\sigma + \mu } \right) + \mu \left( {\beta + \sigma + \mu } \right) } \right] \left\langle {kg\left( k \right) } \right\rangle }}= {R_0} \end{aligned}$$

${R_0}$ is the basic reproductive number of (11). So, we can see that proportion treatment to recuperator is a very effective control strategy, and the bigger the proportion of recuperator to accept treatment, the alcoholism is more difficult to outbreak.

### Proportion prevention

Let $\psi$ be the proportion of susceptible people who understand the harm of alcoholism and refuse to drink, $0< \psi < 1$, then system (11) becomes

19 $$\begin{aligned} \left\{ {\begin{array}{l} \begin{array}{l} \frac{{d{I_k}(t)}}{{dt}} = \frac{{\lambda kg\left( k \right) }}{{\left\langle {kg\left( k \right) } \right\rangle }}\left( {\frac{b}{{b + \mu }} - {I_k}\left( t \right) - {R_k}\left( t \right) } \right) \left( {1 - \psi } \right) \theta \left( t \right) \\ \quad \quad \qquad - \left( {\alpha + \mu } \right) {I_k}(t) + \beta {R_k}(t), \end{array}\\ {\frac{{d{R_k}(t)}}{{dt}} = \alpha {I_k}(t) - \beta {R_k}(t) - \sigma {R_k}(t) - \mu {R_k}(t),} \end{array}} \right. \end{aligned}$$

By the next generation method, we get the basic reproductive number of (19) is

$${\hat{R}_0} = \frac{{b\lambda \left( {\beta + \sigma + \mu } \right) \left( {1 - \psi } \right) \left\langle {kg\left( k \right) \varphi \left( k \right) } \right\rangle }}{{\left( {b + \mu } \right) \left[ {\alpha \left( {\sigma + \mu } \right) + \mu \left( {\beta + \sigma + \mu } \right) } \right] \left\langle {kg\left( k \right) } \right\rangle }}$$

It is easy to know that ${\hat{R}_0} < {R_0}$, and the greater the proportion of susceptible people refuse to drink, the smaller the number of alcoholics.

Next, we are going to compare which strategy is more useful. Transform the form of ${{\tilde{R}}_0}$ and ${\hat{R}_0}$, we have

$${{\tilde{R}}_0} = \frac{{b\lambda \left\langle {kg\left( k \right) \varphi \left( k \right) } \right\rangle }}{{\left( {b + \mu } \right) \left[ {\frac{{\alpha \left( {\sigma + \mu } \right) }}{{\left( {\beta \left( {1 - \varepsilon } \right) + \sigma + \mu } \right) }} + \mu } \right] \left\langle {kg\left( k \right) } \right\rangle }}$$

and

$${{\hat{R}}_0} = \frac{{b\lambda \left\langle {kg\left( k \right) \varphi \left( k \right) } \right\rangle }}{{\left( {b + \mu } \right) \left[ {\frac{{\alpha \left( {\sigma + \mu } \right) }}{{\left( {\beta + \sigma + \mu } \right) \left( {1 - \psi } \right) }} + \frac{\mu }{{\left( {1 - \psi } \right) }}} \right] \left\langle {kg\left( k \right) } \right\rangle }}$$

When $\psi = \varepsilon$, we know that ${{\hat{R}}_0} < {{\tilde{R}}_0}$. That is to say, when the proportion of recuperator to accept treatment is equal to the proportion of susceptible people to refuse drinking alcohol, the strategy of in proportion to prevent susceptible people drinking alcohol will be more effective.

## Sensitivity analysis and numerical simulations

In this section, we perform some sensitivity analysis on the basic reproduction number ${R_0}$ in terms of the parameters. Our simulations take the scale-free networks with degree distribution is $P( k ) = 18{k^{ - 3 }}(2 < \gamma \le 3)$. Let $n=40$, $g( k ) = {k^{{r_1}}}$, $\varphi ( k ) = {k^{{r_2}}}$ and $h( k ) = {k^{{r_3}}}$, $k = 1,2, \ldots ,40$, where ${r_1}$, ${r_2}$ and ${r_3}$ are positive constants. Considering the influence of heavy alcoholics’ relapse and the weight between individuals. We focus on simulate the relapse parameter $\beta$, the weight parameter ${r_1}$ and the nodes’ infectivity parameter ${r_2}$.

**Fig. 2:**
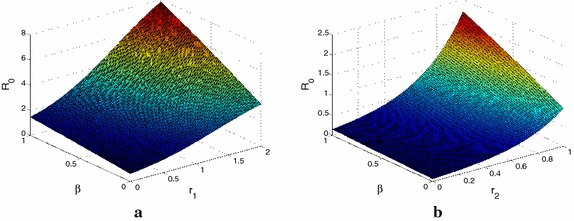
The relationship between the basic reproduction number ${R_0}$ and the parameters on scale-free networks

From Fig. 2, it is clear that ${R_0}$ presents growth trend with the increase of the relapse parameter $\beta$. It means that bigger alcoholism recurrence rate are easy to cause outbreaks of alcoholism. Figure 2a shows that the greater the weight parameter $r_1$ lead to greater ${R_0}$. It means that the greater the link’s weight between two nodes, the easier the alcoholism broke out. Figure 2b shows that ${R_0}$ increases as the nodes’ infectivity parameter $r_2$ increases. That is to say, if a problem alcoholic has big“infectivity“, the alcoholism is more easy to broke out.

Next, we perform some numerical simulations to illustrate our theoretical results of the models (11) and (12), so as to find better control strategies. The parameters are used as $b=0.2$, $\mu =0.04$, $\sigma =0.6$, $\alpha =0.6$, $\beta =0.2$, ${r_1}=1.1$, ${r_2}=1$, ${r_3}= 1.2.$ It is clear that ${r_2}=0$ means no weight model, ${r_2} \ne 0$, ${r_3} = 0$ mean fixed weight model and ${r_2} \ne 0$, ${r_3} \ne 0$ mean adaptive weight model.

**Fig. 3:**
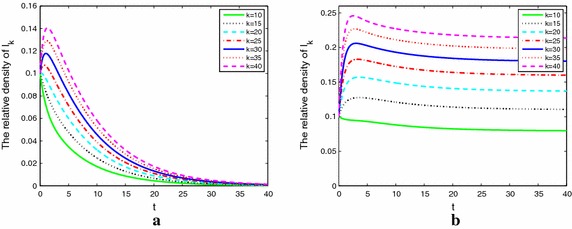
The densities of alcoholics with different degrees and without weight when ${R_0 > 1}$ (**a**) and ${R_0 < 1}$ (**b**)

Figure 3a, b describe nodes without weight with $\lambda =0.05$ and 0.09 respectively, Fig. 3a shows that when ${R_0}=0.8981<1$, the alcoholism dies out quickly. Figure 3b shows that ${R_0}=1.6166>1,$ the problem alcoholics’ population will maintain at a positive stationary level, which implies that the alcoholism will become endemic.

**Fig. 4:**
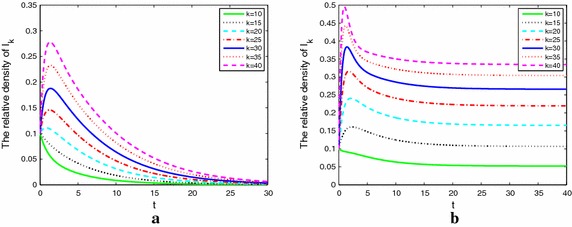
The densities of alcoholics with different degrees and fix weight when ${R_0 > 1}$ (**a**) and ${R_0 < 1}$ (**b**)

Figure 4a, b describe nodes with fixed weight with $\lambda =0.02$ and 0.05 respectively, Fig. 4a shows that when ${R_0}=0.6513<1$, the alcoholism dies out quickly, which implies that the alcohol free equilibrium of (11) is stable. Figure 4b shows that when ${R_0}=1.6283>1$ the problem alcoholics’ population will maintain at a positive stationary level of (11), which implies that the alcoholism will become endemic. Compared with Figs. 3a and 4b, for same parameters, basic reproduction number is 0.8981 and 1.6283, respectively. So we know that ${R_0}$ of model on networks with weights is larger than that on networks without weights.

**Fig. 5:**
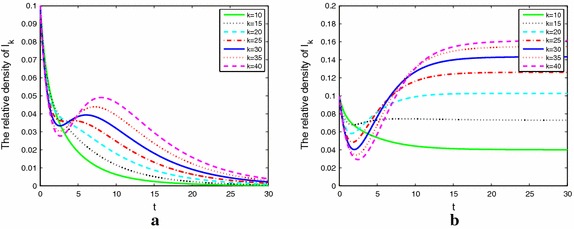
The densities of alcoholics with different degrees and adaptive weight when ${R_0 > 1}$ (**a**) and ${R_0 < 1}$ (**b**)

Figure 5a, b describe nodes with adaptive weight. We know that (12) and (11) have the same ${R_0}$. So we choose the same parameters as Fig. 4. From Fig. 5a, b, we can see that since the adaptivity of weight, the alcoholics rapidly drop first and experience a valley then up to a small peak or dies out. This is caused by the behavior of people’s self-protection awareness. From this point, the adaptive model is more close to the actual situation.

**Fig. 6:**
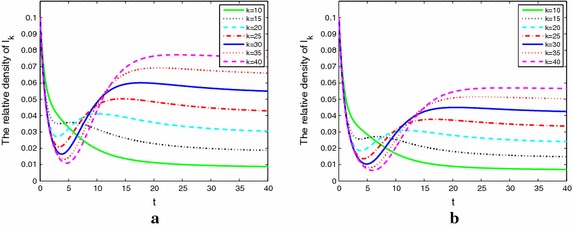
The densities of alcoholics with different adaptive coefficient ${r_3} = 1.4$ in **a** and ${r_3} = 1.5$ in **b**

Figure 6a, b shows the densities of problem alcoholic individuals with different degrees and different the adaptive coefficient ${r_3}.$ We know that the stronger the adaptive coefficient ${r_3},$ the greater the self-protection awareness of susceptible. It lead to alcoholics density maintain in a lower value.

## Conclusion and discussions

In this paper, we proposed a modified SIRS alcoholism model with relapse on weighted networks to study the influences of individual’s contact patterns on drinking dynamics. We construct two complex network models with fixed weight and adaptive weight, respectively. We get the alcoholic propagation threshold ${R_0}$ which determines the propagation dynamics. We also obtain the existence of equilibria of (11). For the model with fixed weight, we prove that when ${R_0} < 1$, the model’s alcohol free equilibrium is global stable and alcoholism will disappear, otherwise, if ${R_0} > 1$, the alcoholism equilibrium is global attractivity and alcoholism will persistence. For the model with adaptive weight, we only make some numerical simulations. By comparing the alcoholism models with no weight, fixed weight and adaptive weight, we have:

1. Fixed weight model have larger propagation threshold than no weight model;
2. The adaptive weight cannot change the propagation threshold, but it can induce the alcoholism to decay quickly;
3. Strong adaptability can inhibit the alcoholism population reach a high level;
4. In order to eliminate alcoholism problem, first we should try to reduce the frequency of interaction between susceptible and problem alcoholic, that is to say decreasing the link’s weight between two nodes, this may be a effective measures. Second, through education or media to spread the dangers of alcohol abuse in order to decrease the relapse $\beta ,$ it is also a very effective measures.

The delay differential equations usually exhibit much more complicated dynamics than ordinary differential equations because the time delay can lead to instability, oscillation, or bifurcation phenomena (Bianca et al. 2015; Bianca and Guerrini 2014). There is a time delay during a susceptible individual becomes the problem alcoholic, so it is more realistic to consider the time delay in the modelling alcoholism process. We can modify (1) to the following model with delay

$$\begin{aligned} \left\{ {\begin{array}{l} {\frac{{d{S_k}(t)}}{{dt}} = b\left( {1 - {S_k}\left( t \right) - {I_k}\left( t \right) - {R_k}\left( t \right) } \right) }\\ \quad \quad \qquad - k{S_k}\left( {t - \tau } \right) \Theta \left( t \right) + \sigma {R_k}(t) - \mu {S_k}\left( t \right) ,\\ {\frac{{d{I_k}(t)}}{{dt}} = k{S_k}\left( {t - \tau } \right) \Theta \left( t \right) + \beta {R_k}\left( t \right) - \alpha {I_k}(t) - \mu {I_k}(t),}\\ {\frac{{d{R_k}(t)}}{{dt}} = \alpha {I_k}(t) - \beta {R_k}\left( t \right) - \sigma {R_k}(t) - \mu {R_k}(t),} \end{array}} \right. \end{aligned}$$

We leave this work for the future.

## References

[CR1] Bansal S, Grenfell B, Meyers LA (2007). When individual behaviour matters: homogeneous and network models in epidemiology. J R Soc Interface.

[CR2] Barrat A, Barthelemy M, Pastor-Satorras R, Vespignani A (2004). The architecture of complex weighted networks. Proc Natl Acad Sci.

[CR3] Barrat A, Barthelemy M, Vespignani A (2004). Modeling the evolution of weighted networks. Phys Rev E.

[CR4] Barrat A, Barthelemy M, Vespignani A (2004). Weighted evolving networks: coupling topology and weight dynamics. Phys Rev Lett.

[CR5] Benedict B (2007). Modeling alcoholism as a contagious disease: how ‘infected’ drinking buddies spread problem drinking. Soc Ind Appl Math News.

[CR7] Bianca C, Guerrini L (2014). Existence of limit cycles in the Solow model with delayed-logistic population growth. Sci World J.

[CR6] Bianca C, Guerrini L, Riposo J (2015). A delayed mathematical model for the acute inflammatory response to infection. Appl Math Inf Sci.

[CR8] Chu XW, Guan JH, Zhang ZZ, Zhou SG (2009). Epidemic spreading in weighted scale-free networks with community structure. J Stat Mech Theory Exp.

[CR9] Chu XW, Zhang ZZ, Guan JH, Zhou SG (2011). Epidemic spreading with nonlinear infectivity in weighted scale-free networks. Phys A.

[CR10] d’Onofrio A (2008). A note on the global behaviour of the network-based SIS epidemic model. Nonlinear Anal Real World Appl.

[CR11] Giraldina TN, Jay KK, Marjolein DW (2015). Alcohol use as a risk factor in infections and healing. Alcohol Res Curr Rev.

[CR12] Glavas MM, Weinberg J (2005) Stress, alcohol consumption and the hypothalamic-pituitary-adrenal axis. In: Yehuda S, Mostofsky DI (eds) Nutrients, stress and medical disorders. Humana Press, NY, pp 165–183

[CR13] Gonzales K, Roeber J, Kanny D (2014). Alcohol-attributable deaths and years of potential life lost—11 states, 2006–2010. Morb Mortal Wkly Rep.

[CR14] Huo HF, Liu YP (2016) Stability of an SAIRS alcoholism model on scale-free networks. J Biol Dyn **(in review)** 10.1080/17513758.2019.1683629 31686626

[CR15] Huo HF, Song NN (2012) Global stability for a binge drinking model with two-stages. Discrete Dyn Nat Soc 2012:1–15

[CR17] Huo HF, Zhu CC (2013) Influence of relapse in a giving up smoking model. Abstr Appl Anal 2013:1–12

[CR16] Huo HF, Wang Q (2014) Modelling the influence of awareness programs by media on the drinking dynamics. Abstr Appl Anal 2014:1–8

[CR18] Lajmanovich A, Yorke JA (1976). A deterministic model for gonorrhea in a nonhomogenous population. Math Biosci.

[CR21] Liu JL, Zhang TL (2011). Epidemic spreading of an SEIRS model in scale-free networks. Commun Nonlinear Sci Numer Simul.

[CR19] Liu JZ, Tang YF, Yang ZR (2004) The spread of disease with birth and death on networks. J Stat Mech 2004(8):P08008

[CR20] Liu MX, Sun GQ, Jin Z, Zhou T (2013). An analysis of transmission dynamics of drug-resistant disease on scale-free networks. Appl Math Comput.

[CR22] Macdonald PJ, Almaas E, Barabasi AL (2005). Minimum spanning trees of weighted scale-free networks. Europhys Lett.

[CR23] Manthey JL, Aidoob AY, Ward KY (2008). Campus drinking: an epidemiological model. J Biol Dyn.

[CR24] Mushayabasa S, Bhunu CP (2011). Modelling the effects of heavy alcohol consumption on the transmission dynamics of gonorrhea. Nonlinear Dyn.

[CR25] Room R, Babor T, Rehm J (2005). Alcohol and public health. Lancet.

[CR26] Sanchez F, Wang XH, Castillo-Chavez C, Gorman DM, Gruenewald PJ (2007). Drinking as an epidemica—a simple mathematical model with recovery and relapse. Ther Guide Evid Based Relapse Prev.

[CR27] Saunders JB, Aasland OG, Amundsen A, Grant M (1993). Alcohol consumption and related problems among primary health care patients: WHO collaborative project on early detection of persons with harmful alcohol consumption. Addiction.

[CR28] Sharma S, Samanta GP (2015). Analysis of a drinking epidemic model. Int J Dyn Control.

[CR29] Sumana P, Tasha B, Llhem M (2015). Impact of alcohol abuse on the adaptive immune system. Alcohol Res Curr Rev.

[CR30] van den Driessche P, Watmough J (2002). Reproduction numbers and sub-threshold endemic equilibria for compartmental models of disease transmission. Math Biosci.

[CR31] Varga RS (2000). Matrix lterative analysis.

[CR33] Wang Y, Jin Z, Yang ZM, Zhang ZK, Zhou T, Sun GQ (2012). Global analysis of an SIS model with an infective vector on complex networks. Nonlinear Anal Real World Appl.

[CR32] Wang XY, Huo HF, Kong QK, Shi WX (2014) Optimal control strategies in an alcoholism model. Abstr Appl Anal 2014:1–18

[CR34] Wechsler H (2000) Binge drinking on America’s college campuses: findings from the harvard school of public health college alcohol study, vol 1. Harvard School of Public Health, pp 3–5

[CR35] Wechsler H, Nelson TF (2008). Focusing attention on college student alcoholcon sumption and the environmental conditions that promoteit. J Stud Alcohol Drugs.

[CR36] Yorke JA (1967). Invariance for ordinary differential equations. Math Syst Theory.

[CR37] Zhang JP, Jin Z (2011). The analysis of an epidemic model on networks. Appl Math Comput.

[CR38] Zhu GH, Fu XC, Chen GR (2012). Global attractivity of a network-based epidemic SIS model with nonlinear infectivity. Commun Nonlinear Sci Numer Simul.

[CR39] Zhu GH, Fu XC, Chen GR (2012). Spreading dynamics and global stability of a generalized epidemic model on complex heterogeneous networks. Appl Math Model.

[CR40] Zhu GH, Chen GR, Xu XJ, Fu XC (2013). Epidemic spreading on contact networks with adaptive weights. J Theor Biol.

